# Withaferin-A—A Natural Anticancer Agent with Pleitropic Mechanisms of Action

**DOI:** 10.3390/ijms17030290

**Published:** 2016-03-04

**Authors:** In-Chul Lee, Bu Young Choi

**Affiliations:** 1 Department of Cosmetic science, Seowon University, Cheongju, Chungbuk 361-742, Korea; lic9418@seowon.ac.kr; 2 Department of Pharmaceutical Science & Engineering, Seowon University, Cheongju, Chungbuk 361-742, Korea

**Keywords:** withaferin-A, phytochemical, cancer, chemoprevention, chemotherapy

## Abstract

Cancer, being the second leading cause of mortality, exists as a formidable health challenge. In spite of our enormous efforts, the emerging complexities in the molecular nature of disease progression limit the real success in finding an effective cancer cure. It is now conceivable that cancer is, in fact, a progressive illness, and the morbidity and mortality from cancer can be reduced by interfering with various oncogenic signaling pathways. A wide variety of structurally diverse classes of bioactive phytochemicals have been shown to exert anticancer effects in a large number of preclinical studies. Multiple lines of evidence suggest that withaferin-A can prevent the development of cancers of various histotypes. Accumulating data from different rodent models and cell culture experiments have revealed that withaferin-A suppresses experimentally induced carcinogenesis, largely by virtue of its potent anti-oxidative, anti-inflammatory, anti-proliferative and apoptosis-inducing properties. Moreover, withaferin-A sensitizes resistant cancer cells to existing chemotherapeutic agents. The purpose of this review is to highlight the mechanistic aspects underlying anticancer effects of withaferin-A.

## 1. Introduction

Despite the development of a wide variety of anticancer drugs, the global incidence of various cancers, and the mortality thereof, is still on the rise. The number of cancer-related deaths is presumed to increase by two-fold in the next 50 years [1]. The increasing trend of chemotherapy resistance and frequent relapse of certain tumors make it more complicated to eradicate cancer by using conventional chemotherapeutic agents. Thus, there is always a need for developing new anticancer therapies to combat this dreadful disease. Plants have always been at the fore-front of new drug discovery. A large number of plant constituents, commonly known as phytochemicals, have gained the status of clinically used modern medicines, while many others have served as potential lead compounds for designing novel therapeutics [2]. In fact, plants are natural chemical factories for synthesizing chemicals with structural diversity, such as steroids, terpenoids, flavonoids, alkaloids, *etc*. Many of these plant secondary metabolites are biologically active, and can interact directly or indirectly with various cellular components, especially proteins and lipids, thereby reversing the altered functions of cancer cells [2,3]. Since the growth and development of tumors are triggered by oxidative stress and chronic inflammation, phytochemicals with antioxidative and anti-inflammatory properties are thought to play important roles in the prevention and/or treatment of cancer. Until now, numerous phytochemicals have been examined for their potential anticancer properties by using a diverse array of preclinical experimental models.

**Figure 1:**
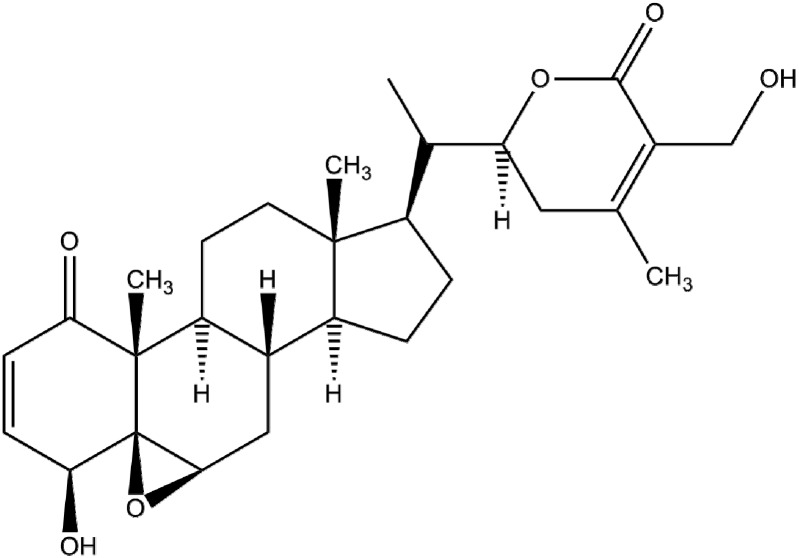
Structure of withaferin-A.

A large number of laboratory-based studies have demonstrated the anticancer effects of a medicinal plant, *Withania somnifera* (L.) Dual (Solanaceae), commonly known as Ashwagandha, Indian ginseng or Indian winter cherry [4]. Different parts of the plant have long been used in a wide variety of Ayurvedic formulations. However, systematic scientific research on the plant begun in the 1950s [5]. The anticancer property of *W. sominefra* was first addressed by Devi and colleagues [6], who showed that intraperitoneal (i.p.) administration of alcoholic root extract of the plant completely regressed the growth of sarcoma-180 cells inoculated in nude mice. Subsequent studies have demonstrated that the hydroalcoholic root extract of the plant attenuated 20-methylcholathrene (20MC)-induced development of fibrosarcoma in Balb/C mice when administered intraperitoneally [7] or by gavage [8]. Davis and Kuttan [9] have further reported the inhibitory effect of *W. somnifera* extract on chemically induced mouse skin tumor development. According to a recent study, daily administration of root extract of the plant markedly reduced methylnitrosourea-induced rat mammary tumorigenesis [10]. These anticancer properties of Ashwagandha are attributable to withanolides, a class of bioactive constituents isolated from *W. sominifera*. Withaferin-A (4β,5β,6β,22*R*)-4,27-Dihydroxy-5,6-22,26-diepoxyergosta-2,24-diene-1,26-di one, structure shown in Figure 1), is the first antitumor withanolide that was isolated from leaves of the plant back in 1967 [8].

Pretreatment of Syrian golden hamsters with withaferin-A significantly reduced 7,12-dimethylbenz(α)anthracene (DMBA)-induced frequency of micronucleated polychromatic erythrocytes and chromosomal aberrations, indicating the antigenotoxic effect of withaferin-A [11]. A great deal of research has been conducted to elucidate the molecular mechanisms underlying anticancer effects of withaferin-A. This mini-review addresses the biochemical basis of antitumor potential of withaferin-A.

## 2. Effects of Withaferin-A on Multiorgan Carcinogenesis—Evidence from Animal Model Studies

Systematic research on the evaluation of anticancer activities of withaferin-A was started around the 1970s [12]. Since then, a large number of studies have demonstrated the ability of withaferin-A to suppress the *in vivo* growth of various human cancer cells’ xenograft tumors as well as experimentally induced carcinogenesis in different rodent models [4]. Here, we discuss the chemopreventive effects of withaferin-A on organ-specific carcinogenesis *in vivo*.

### 2.1. Sarcoma and Ascites Tumor

In a pioneering study, Shohat and colleagues reported that i.p. administration of withaferin-A reduced the growth of Ehrlich ascites tumor with an LD_50_ value of 400 mg/kg in Swiss albino mice [12] and prolonged the survival of mouse sarcoma (S-180) and ascites tumor-bearing mice [13]. Electron microscopic analysis of implanted tumors revealed that treatment with withaferin-A altered the spindle microtubule organization, suggesting a probable mechanism of tumor growth inhibition by the compound. Subsequent studies have reported that withaferin-A exerted inhibitory effects on the growth of mouse ascites carcinoma [14], and fibrosarcoma [15] cells in mice. Likewise, the *in vivo* growth of soft tissue sarcoma cells implanted in female SCID (severely combined immunodeficient) mice was inhibited by i.p. administration of withaferin-A, which reduced cell proliferation and induced apoptosis via degradation of vimentin [16]. Contrary to these reports, oral administration of withaferin-A failed to inhibit the growth of HT1080 fibrosarcoma cells xenograft tumors in mice [17].

### 2.2. Prostate Cancer

Yang *et al.* first reported the mechanism-based anticancer activity of withaferin-A, which reduced the growth of human prostate cancer (PC3) cells tumor xenograft in nude mice by blocking the tumor angiogenesis and inducing intratumoral apoptosis [16]. According to this study, i.p. administration of withaferin-A caused regression of implanted tumor cells by decreasing the expression of angiogenesis marker CD31, inducing the expression of proapoptotic protein Bax, and activating caspase-3 via inhibition of nuclear factor-κB (NF-κB) signaling pathway [18]. In a separate study, intratumoral administration of withaferin-A arrested PC3 cells’ xenograft tumor growth in mice by inducing tumor cell death via upregulation of prostate apoptosis response-4 (Par-4).

### 2.3. Gynecological Cancer

The *in vivo* growth of various human gynecological cancer cells was also attenuated by withaferin-A. The compound inhibited the growth of human breast cancer (MDA-MB-231) cells injected subcutaneously to nude mice [19] and 4T mouse mammary tumor cells implanted orthotopically into Balb/c mice [20]. While the tumor growth suppression in the former study was associated with reduced expression of proliferating cell nuclear antigen (PCNA), the later study demonstrated that the tumor regression was linked to increased phosphorylation of vimentin at serine-56 residue. In a clinically relevant transgenic animal model of breast cancer in MMTV-neu mice, treatment with withaferin-A failed to alter the incidence and multiplicity of mammary tumors, but resulted in a 50% inhibition in the weight of palpable tumors and a 95.14% decrease in the mean area of microscopic invasive carcinoma as compared to the control group [21]. The inhibition of mammary tumor growth in this study was associated with increased apoptosis and deregulated tumor cell metabolism. Treatment with withaferin-A intervened with mitochondrial respiration via inhibition of complex III activity, and diminished the levels of intermediates in glucose metabolism pathway. However, tumor cell proliferation and angiogenesis remained unaffected upon treatment of withaferin-A [21]. According to Munagala *et al.* [22], the compound decreased the volume of human cervical cancer (Caski) cells xenograft tumor in athymic nude mice by 70%, which was associated with the inhibition of human papilloma virus E6/E7 oncogenes and the induction of p53 tumor suppressor gene expression. A more recent study demonstrated that administration of withaferin-A attenuated *in vivo* growth and metastasis of orthotopic ovarian tumors generated by injecting human ovarian cancer (A2780) cells in nude mice [23].

### 2.4. Melanoma

Withaferin-A has been reported to suppress mouse melanoma (B16F1) tumor growth *in vivo* [24]. In another skin cancer xenograft model using 92.1 uveal melanoma cells, about 29% of mice treated with withaferin-A exhibited a complete clinical response, while 43% of the animals showed cancer progression upon discontinuation of treatment [25]. Li and colleagues [26] have recently reported that withaferin-A reduced the tumor multiplicity, though not the incidence, of DMBA-initiated and 12-*O*-tetradecanoylphorbol-13-acetate (TPA) promoted mouse skin tumor formation partly by blocking the expression of acetyl CoA carboxylase-1 (ACC1) and the activation of activator protein-1 (AP-1).

### 2.5. Thyroid Cancer

Administration of withaferin-A to medullary thyroid cancer (DRO81-1) cells xenograft tumor-bearing nude mice showed significant inhibition of tumor growth and metastasis. This antitumor effect was associated with a marked decrease in total and phosphorylated RET and pro-caspase-3, suggesting the induction of apoptosis by withaferin-A in xenografted tumors [27]. Likewise, the increased expression of apoptotic markers, such as Bax, p27, cell cycle and apoptosis regulatory protein-1 (CARP1) was associated with the inhibition of mouse mesothelioma xenograft tumor growth in Balb/c mice upon i.p. administration of withaferin-A [28].

### 2.6. Gastrointestinal Cancer

Administration of withaferin-A (20 mg/kg body weight) by gavage suppressed DMBA-induced oral carcinogenesis in hamsters, at least in part, by conferring antioxidant defense through the inhibition of lipid peroxidation and induction of cytoprotective enzymes, and promoting tumor cell apoptosis [29,30,31]. Withaferin-A also inhibited the growth of pancreatic cancer (Panc-1) cells xenograft tumor by 30% and 58%, when given at 3 or 6 mg/kg body weight, respectively [32]. We have recently reported that intraperitoneal administration of withaferin-A (2 mg/kg body weight) for six weeks significantly inhibited the volume and weight of human colon cancer (HCT116) cells xenograft tumors in BALB/c mice [33]. The tumor growth suppression was associated with decreased cell proliferation as revealed by reduced expression of proliferating cell nuclear antigen (PCNA) in withaferin-A-treated xenograft tumor as compared with that of control animals [33].

### 2.7. Other Cancers

Several other studies have reported the inhibitory effects of withaferin-A on the growth of mouse mesothelioma [28] and human pancreatic cancer (Panc-1) cells xenograft tumor growth in mice [32], which involved the induction of intra-tumoral cell death. AshwaMAX (40 mg/kg/day), a formulation of concentrated withaferin-A, was given by gavage to female nude mice receiving orthotopically administered human parietal-cortical glioblastoma (GBM2) cells transduced with lentiviral vectors expressing Green Fluorescent Protein (GFP) or firefly luciferase fusion proteins, and the growth of intracranial glioblastoma xenografts tumor was monitored by bioluminescence imaging (BLI). AshwaMAX (40 mg/kg per day) significantly reduced BLI signals indicating decreased tumor growth but after 30 days of treatment the BLI signal was increasing, suggesting the onset of resistance. Moreover, AshwaMAX inhibited the neurosphere cultures (U87-MG, GBM2 and GBM39) at IC_50_ values of 1.4, 0.19 and 0.22 µM, respectively. Incubation with withaferin-A also attenuated neurosphere growth of U87-MG, GBM2 and GBM39 glioblastoma cells with IC_50_ values of 0.31, 0.28 and 0.25 µM, respectively [34].

## 3. Biochemical Basis of Anticancer Effects of Withaferin-A

Since the neoplastic transformation of cells involves oxidative and/or inflammatory stress-induced modifications of cellular macromolecules, such as DNA, RNA and proteins, and subsequent dysfunction of cellular biochemical pathways, the tumorigenesis process can be prevented by relieving cells from oxidative stress load and inflammatory insults.

**Figure 2:**
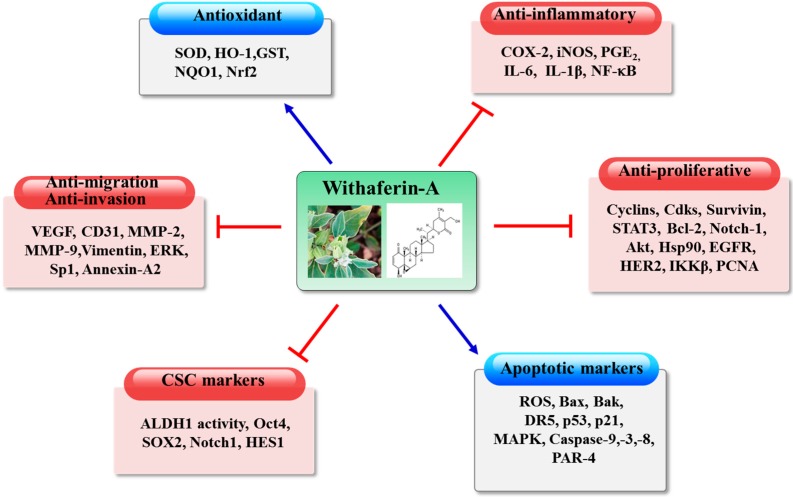
Potential targets of the beneficial effects of withaferin-A in Cancer development. Withaferin-A exhibits therapeutic potential for cancer chemoprevention and may aid in the prevention of age-related disorders, such as anti-oxidant and inflammation disease. Antioxidant: SOD (superoxide dismutase), HO-1 (heme oxygenase-1), GST (glutathione s-transferase), NQO1 (NAD (P)H dehydrogenase, quinone 1), Nrf2 (nuclear factor E2-related factor 2), Anti-inflammatory : COX-2(Cyclooxygenase 2), iNOS (Nitric oxide synthases), PGE2(prostaglandin E2), IL-6(Interleukin 6), IL-1β(Interleukin 1β), NFκB (nuclear factor κ light chain enhancer of activated B cells), Anti-proliferative : CDKs (Cyclin-dependent kinases), STAT3 (Signal transducer and activator of transcription 3), Bcl2 (B-cell lymphoma 2), Notch-1 (Notch homolog 1, translocation-associated), AKT(serine/threonine-specific protein kinase), Hsp90(heat shock protein 90), EGFR(epidermal growth factor receptor), HER2 (human epidermal growth factor receptor 2), IKKb (inhibitor of nuclear factor κB kinase subunit β), PCNA (Proliferating cell nuclear antigen), Apoptosis markers: ROS (reactive oxygen species), Bax (BCL2-associated X protein), Bak (Bcl-2 homologous antagonist/killer), DR5 (Death receptor 5), MAPK(Mitogen-activated protein kinases), PAR-4 (Protease-activated receptor 4)., CSC marker : ALDH1 (Aldehyde dehydrogenase 1), Oct4 (octamer-binding transcription factor 4 ), SOX2 (SRY (sex determining region Y) -box 2), Notch1 (Notch homolog 1, translocation-associated), HES1 (Transcription factor HES1), Anti-migration, anti-invasion: VEGF (Vascular endothelial growth factor), CD31 (cluster of differentiation 31), MMP (Matrix metalloproteinases), ERK (extracellular-signal-regulated kinases), Sp-1 (specificity protein 1).

Tumor cells acquire biochemical features, marked as a hallmark of cancer, that include loss of density-dependent inhibition of growth and autonomous ability of cell proliferation, altered cellular metabolism, deregulated cell cycle, evasion from apoptosis, increased tumor angiogenesis, acquiring invasion and metastasis potential, and the development of immune escape mechanisms [35]. In fact, tumors grow as an aggregate and collaboration of diverse host-derived cells, such as inflammatory immune cells and stromal cells, thereby creating a microenvironment within the growing tumor mass. Intra-tumoral inflammation acts as the trigger for attaining many of the hallmarks of cancer [36]. Thus, intervention with tumor-specific biochemical processes is the mechanistic basis of the anticancer effects of withaferin-A (Figure 2). These include: (1) reinforcement of cellular antioxidant and/or detoxification system [29,30,31]; (2) suppression of inflammatory pathways [37,38,39]; (3) selective inhibition of tumor cell proliferation and induction of apoptosis; (4) suppression of tumor angiogenesis; (5) blockade of epithelial-to-mesenchymal transition (EMT), tumor invasion, and metastasis; (6) alteration of tumor cell metabolism; (7) immunomodulation; and (8) eradication of cancer stem cells.

### 3.1. Effects of Withaferin-A on Cytoprotective Enzymes

A battery of cytoprotective enzymes with antioxidative and detoxification potential are endogenously induced in response to mild oxidative stress to protect cellular macromolecules from oxidative and/or covalent modifications, thereby regulating cellular homeostasis. Major cytoprotective enzymes include, but are not limited to, NAD(P)H-quinone oxidoreductase-1 (NQO1), superoxide dismutase (SOD), catalase (CAT), glutathione peroxidase (GPX), and heme oxygenase-1 (HO-1). Transcriptional activation of genes encoding these cytoprotective enzymes involve the activation of a redox-sensitive transcription factor NF-E2-related factor-1 (Nrf2), which normally resides in cytoplasm by forming an inactive complex with its inhibitory protein Kelch-like ECH associated protein-1 (Keap1). Genetic ablation of several of these cytoprotective enzymes or that Nrf2 have been reported to increase the susceptibility of animals to carcinogens in various mouse model of carcinogenesis [40]. Thus, the induction of cytoprotective enzymes via activation of Nrf2 by phytochemicals would protect against oxidative damage of cellular components, thereby preventing tumor development. In fact, numerous Nrf2 activators have been shown to inhibit experimental carcinogenesis in a diverse *in vitro* and *in vivo* model of studies [40].

The antioxidant activity of withaferin-A was first reported by Bhattacharya *et al.* [41], who showed that treatment with withaferin-A increased SOD, CAT and GPX activity in rat brain frontal cortex and striatal concentrations. Treatment of mice with withaferin-A restored acetaminophen-induced depletion of hepatic glutathione (GSH), and increased the hepatic up-regulation of Nrf2, glutamyl cysteine ligase-catalytic subunit (GCLc) and NQO1, and down-regulated the production of inflammatory cytokines, interleukin (IL)-6 and IL-1β, thereby protecting liver from injury [42]. However, detailed mechanisms underlying Nrf2 activation and cytoprotective enzyme induction by withaferin-A is still unclear. Although the activation of Nrf2 and its target genes is considered as a pragmatic approach for cancer prevention, the induction of Nrf2-mediated signaling provides survival benefit to cancer cells, and hence selective inhibition of Nrf2 and suppression of antioxidant enzyme expression in tumors are thought to enhance tumor cell death [43]. Thus, the complete inhibition of oral squamous cell carcinoma formation by withaferin-A in a model of DMBA-induced hamster oral carcinogenesis is associated with the ability of the compound to return the level/activity of antioxidants, such as GSH or glutathione-*S*-transferase (GST), which is elevated upon DMBA-treatment, to near normal levels [29,44]. However, these studies lack the effect of withaferin-A on Nrf2 status. A recent study demonstrated that withaferin-A induced the expression of Nrf2, and its target gene products, such as HO-1 and NQO1, in human colorectal cancer cells by generating ROS and activating c-Jun-N-terminal kinase (JNK) as a regulatory kinase upstream of Nrf2. However, the Nrf2 activation and NQO1 expression by withaferin-A in colorectal cells resulted in the induction of cell death, rather than cell survival, via blockade of proteasomal degradation of TAp73 tumor suppressor gene. Since withaferin-A harbors α,β-unsaturated carbonyl moieties in its structure, it is likely that the compound can act as a Michael acceptor and a thiol modifier. In fact, several studies have already shown that withaferin-A can modify cysteine thiols of several key cell signaling molecules [45,46], which will be discussed in subsequent sections. Thus, it would interesting to further investigate if withaferin-A can modify Keap1-cysteine residues, and thereby regulate Nrf2 activation in normal and cancerous cells, and their implication in tumorigenesis.

### 3.2. Anti-Inflammatory Effects of Withaferin-A

Inflammation is the seventh hallmark of cancer. Elevation of various pro-inflammatory mediators, such as prostaglandins (PGs), nitric oxide (NO), and cytokines in the tumor microenvironment or in persistently inflamed tissues contribute to carcinogenesis [47,48]. PGs are the products of arachidonic acid metabolism by the enzyme cyclooxygenase (COX), whereas NO is generated as a result of increased expression and activity of inducible nitric oxide synthase (iNOS). Cytokines are produced by the inflammatory immune cells within a tumor microenvironment. Elevated expression and/or activity of COX-2 and iNOS, and the resultant generation of pro-inflammatory mediators have been implicated in carcinogenesis. Thus, inhibition of aberrant expression of COX-2 and iNOS may prevent the promotion and progression of cancer [49]. Withaferin-A inhibited the expression of iNOS at both protein and mRNA levels in lipopolysaccharide (LPS)-stimulated murine macrophage RAW264.7 cells by blocking the phosphorylation of Akt and extracellular signal-regulated kinases (ERKs) via inhibition of IκB phosphorylation and subsequent nuclear factor-κB (NF-κB) activation [50]. This group further demonstrated that withaferin-A inhibited LPS-induced COX-2 mRNA and protein expression and PGE_2_ production in BV2 murine microglial cells, partly by blocking the phosphorylation of p38 mitogen-activated protein (MAP) kinase and JNK via blockade of nuclear translocation and DNA binding of signal transducer and activator of transcription (STAT)-1 and -3 [51]. Martorana *et al.* also reported that both pre- and post-treatment of astrocytes with withaferin-A attenuated LPS-induced production of tumor necrosis factor-α (TNFα), and the expression of COX-2 and iNOS by blocking the NF-κB activity [52]. In another study, withaferin-A diminished Helicobacter pylori-mediated production of IL-8 and the activation of NF-κB in human gastric epithelial AGS cells [53]. Since Helicobacter pylori-induced gastritis is a predisposing factor for the development of stomach tumors, withaferin-A may be useful in preventing gastric carcinogenesis.

WFA inhibited lipopolysaccharide (LPS)-induced HMGB1 release and HMGB1-mediated barrier disruption, expression of cell adhesion molecules (CAMs) and adhesion/transendothelial migration of leukocytes to human endothelial cells. WFA also suppressed acetic acid-induced hyperpermeability and carboxymethylcellulose-induced leukocytes migration *in vivo*. Further studies revealed that WFA suppressed the production of interleukin 6, tumor necrosis factor-α (TNF-α) and activation of nuclear factor-κB (NF-κB) by HMGB1. Collectively, these results suggest that WFA protects vascular barrier integrity by inhibiting hyperpermeability, expression of CAMs, adhesion and migration of leukocytes, thereby endorsing its usefulness as a therapy for vascular inflammatory diseases.

Treatment of human umbilical vein endothelial cells (HUVECs) with withaferin-A resulted in a marked decrease in LPS-induced release of high mobility group box 1 protein (HMGB1) and subsequent blockade of barrier disruption and cell adhesion molecules (CAMs) expression. Withaferin-A also attenuated acetic acid-induced hyperpermeability and carboxymethylcellulose-induced leukocytes migration in mice. In addition, withaferin-A attenuated the production of IL-6, TNF-α, and the activation of NF-κB in HMGB1-stimulated HUVECs [37]. The compound reduced the shedding of endothelial cell protein C receptor (EPCR), a mediator of vascular inflammation, in TPA-stimulated HUVECs by blocking the phosphorylation of MAP kinases [54].

### 3.3. Antiproliferative Effects of Withaferin-A

The inhibition of cell proliferation and induction of apoptosis are the prime mechanisms underlying anticancer effects of withaferin-A. Table 1 summarizes the molecular targets of withaferin-A in relation to its antiproliferative and apoptosis inducing activity. Withaferin-A inhibited proliferation of various cancer cells mainly by inducing G2/M phase cell cycle arrest [22,55,56,57,58,59]. The compound induced G2/M phase arrest in human prostate cancer (PC-3 and DU-145) cells via increased phosphorylation of Wee-1, histone H3, p21, and Aurora kinase-B, and downregulation of cyclins (A2, B1 and E2) and a decrease in Cdc2 (Tyr15) phosphorylation. This study also demonstrated that withaferin-A decreased the levels of phosphorylated Chk1 (Ser345) and Chk2 (Thr68), suggesting that the activation of Cdc2 leads to cell cycle arrest in the M phase and initiation of mitotic catastrophe [58]. Incubation of temozolomide-resistant glioblastoma multiforme (U251TMZ and U87TMZ) cells with withaferin-A also resulted in G2/M phase cell cycle arrest as revealed by increased expression of cyclin B1, which was accompanied by the depletion of tyrosine kinase cell surface receptors c-Met, epidermal growth factor receptor (EGFR), and Her-2, and reduced phosphorylation of cell survival kinases, such as Akt, mTOR and p70 S6K [56]. Likewise, withaferin-A induced cell cycle arrest in human osteosarcoma (MG63 and U20S) cells at the G2/M phase, which was associated with the inhibition of cyclin-B1 and -A, Cdk2 and p-Cdc2 (Tyr15) expression and an increase in the levels of p-Chk1 (Ser345) and p-Chk2 (Thr68) [57].

The induction of G2/M phase cell cycle arrest by withaferin-A was also noted in human ovarian cancer (CaOV3 and SKOV3) [60] and colorectal cancer (HCT116 and SW480) cells [55]. The former study reported that the compound downregulated the cell survival mediated through Notch-3/Akt/Bcl-2 signaling, thereby causing caspase-3 activation leading to the induction of apoptosis [60]. The latter study, on the other hand, demonstrated that withaferin-A delayed mitosis by blocking the function of spindle assembly checkpoint through proteasomal degradation of Mad2 and Cdc20, which are important constituents of spindle checkpoint complex [55]. Moreover, the proliferation of human colon cancer cells by withaferin-A was attenuated due to the decreased Notch-1 expression [61].

The compound arrested the proliferation of human non-small cell lung cancer (A549) cells at G0/G1 phase, which was associated with the inhibition of phosphatidyl inositol-3 kinase (PI3K)/Akt signaling and reduced the expression of Bcl-2 [62]. Withaferin-A elicited potent antiproliferative activity against pancreatic cancer (Panc-1, MiaPaCa2 and BxPc3) cells by blocking heat shock protein (Hsp)-90 chaperone activity through interaction with the C-terminus of Hsp90, thereby inducing degradation of Hsp90 client proteins, such as Akt, Cdk4 and glucocorticoid receptor [32]. Withaferin-A inhibited the survival of human and murine B cell lymphomas in culture, and attenuated the growth of syngeneic-graft lymphoma cells xenograft tumor *in vivo* without affecting other proliferative tissues by blocking NF-κB nuclear translocation in diffuse large B cell lymphomas. As a result, the compound negated B cell receptor signaling and cell cycle progression, at least in part, by inhibiting the activity of heat shock protein (Hsp)-90, which is a regulator of many critical kinases and cell cycle regulators [63].

Withaferin-A-mediated G2/M phase cell cycle arrest in cervical cancer (Caski) cells was associated with decreased expression of cyclin B1, p34 (Cdc2) and proliferating cell nuclear antigen (PCNA), and diminished phosphorylation of STAT3 at tyrosine 705 and serine 727 residues [22]. Antony *et al.* demonstrated that withaferin-A selectively induced G2 phase and mitotic arrest, as evident by disruption of spindle assembly, in MCF-7, SUM159, and SK-BR-3 cells, but not in normal human mammary epithelial (MCF-10A) cells by markedly reducing the protein levels of β-tubulin. However, the C6,C7-epoxy analogs (withanone and withanolide-A) failed to exhibit such effects. According to this study, nuclear magnetic resonance analysis showed that the A-ring enone of withaferin-A, but not of withanone or withanolide A, formed irreversible nucleophilic addition due to its high reactivity with cysteamine. Direct covalent binding of withaferin-A to cysteine-303 residue of β-tubulin in MCF-7 cells was detected by Mass spectroscopy. In addition, molecular docking studies revealed that withaferin-A interacted with the binding site, exemplified by the organization of hydrophobic floor and wall having a hydrophilic entrance, on the surface of β-tubulin [64]. Treatment of triple negative breast cancer (MDA-MB-231 and BT20) cells with withaferin-A reduced cell proliferation through denaturation and proteasomal degradation of pro-survival factors heat shock factor 1 (HSF1) and breast cancer susceptibility gene 1 (BRCA1) [59].

### 3.4. Induction of Tumor Cell Apoptosis by Withaferin-A

#### 3.4.1. Involvement of ROS in Withaferin-A-Induced Apoptosis

Although several studies have previously reported that generation of ROS contributes to withaferin-A-induced cell death [4], the first concrete proof of the involvement of ROS in apoptosis induction by the compound in human breast cancer (MDA-MB-231 and MCF-7) cells was provided by Hahm *et al.* [75]. According to their study, withaferin-A caused ROS production and apoptosis induction in cancer cells, but not in a normal human mammary epithelial (HME) cells. Withaferin-A-mediated ROS production and DNA fragmentation was attenuated by ectopic expression of Cu,Zn-superoxide dismutase in both MDA-MB-231 and MCF-7 cells. This study also showed that withaferin-A-induced ROS generation was accompanied by the blockade of oxidative phosphorylation and inhibition of complex III activity, resulting in the decrease of mitochondrial membrane potential. Moreover, withaferin-A-mediated cell death was associated with activation of Bax and Bak in MDA-MB-231 and MCF-7 cells [75].

#### 3.4.2. Intrinsic and Extrinsic Mechanisms of Apoptosis Induction by Withaferin-A

Withaferin-A induced apoptotic cell death with various IC_50_ values ranging from 1.8 to 6.1 μM in four different melanoma cells. The susceptibility of cells toward withaferin-A-induced apoptosis was correlated with low Bcl-2/Bax and Bcl-2/Bim ratios. Withaferin-A activated the mitochondrial apoptosis pathway that was associated with Bcl-2 down regulation, Bax up regulation, caspase 9 and caspase-3 activation through cytochrome c release into the cytosol resulting in the cleavage of DNA. All these intrinsic pathways of apoptosis induction required ROS production by withaferin-A [70]. Likewise, withaferin-A-induced decrease in cell viability in leukemia cells was mediated through the externalization of phosphatidylserine, a time-dependent increase in Bax/Bcl-2 ratio, the loss of mitochondrial transmembrane potential, cytochrome c release, activation of caspases-9 and -3 activation, and arresting of cells in sub-G0 phase [77]. A recent study demonstrated that withaferin-A enhanced hyperthermia-induced tumor cell death in cervical cancer (HeLa) cells in a mitochondria-mediated pathway. As compared to the treatment with withaferin-A or hyperthermia alone, exposure of hyperthermia-stimulated HeLa cells to withaferin-A resulted in decreased intracellular GSH/GSSG ratio and caspase-3 activation, which was significantly inhibited by a cell permeable glutathione precursor. This combination treatment led to a decrease in mitochondrial transmembrane potential, increase in expression of pro-apoptotic Bcl-2-family proteins (e.g., tBid and Noxa), and the downregulation of anti-apoptotic proteins (e.g., Bcl-2 and Mcl-1 [71]).

Treatment of human renal cancer (Caki) cells with sub-toxic concentrations of withaferin-A potentiated tumor necrosis factor-related apoptosis-inducing ligand (TRAIL)-induced apoptosis, without affecting the viability of human normal mesangial cells. Withaferin-A generated ROS and up-regulated the expression of death receptor-5 (DR5) in a CCAAT-enhancer binding protein (C/EBP)-homologous protein (CHOP)-dependent manner. Pretreatment with *N*-acetyl cysteine (NAC) or catalase abrogated withaferin-A-induced up-regulation of both CHOP and DR5 [68]. The induction of apoptosis in human head and neck carcinoma (MC3 and HN22) cells by withaferin-A was accompanied by upregulation of Bim, truncated Bid (t-Bid), cleavage of caspase-8, -3 and poly ADP ribose polymerase (PARP), and the elevated expression of DR5, suggesting an extrinsic pathway of apoptosis induction by this compound [67]. Mechanistically, the transcriptional activation of DR5 was mediated through withaferin-A-induced phosphorylation of ERK1/2 and ribosomal S6 kinase, and the induction of ETS-like transcription factor 1 (Elk1) and CHOP [78].

Withaferin-A-induced apoptosis in human leukemia (HL-60) cells was associated with ROS-mediated activation of both intrinsic and extrinsic pathways as shown by the induction of Bax, decreased mitochondrial membrane potential and release of cytochrome-c, activation of caspase-9, -3 and -8, and the cleavage of PARP. These effects of withaferin-A were mediated through activation of TNFα receptor-1 (TNFR1) and inactivation of NF-κB transcription factor [69]. Similarly, the induction of apoptosis of U251TMZ and U87TMZ cells by withaferin-A was mediated through the activation of both extrinsic and intrinsic pathways through ROS-dependent manner [56].

#### 3.4.3. Withaferin-A Alters the Expression of pro- and Anti-Apoptotic Proteins

Exposure of human breast cancer (MDA-MB-231 and MCF-7) cells with varying concentrations of withaferin-A resulted in the apoptosis induction characterized by the cleavage of poly-(ADP-ribose)-polymerase, and the condensation and fragmentation of DNA without inducing similar effects in MCF-10A cells [19]. The apoptosis induction in these cells was associated with decreased expression of Bcl-2 and increased expression of Bim-S and Bim-EL that was negated upon silencing with forkhead box O3a (FOXO3a), a transcriptional regulator of Bim [79]. Moreover, incubation of MDA-MB-231 and MCF-7 cells in presence of withaferin-A resulted in the suppression of X-linked inhibitor of apoptosis (XIAP), cIAP-2, and survivin protein levels. Ectopic overexpression of XIAP, survivin, and cIAP-2 in both cells abrogated withaferin-induced apoptosis [72].

#### 3.4.4. Withaferin-A-Induces Apoptosis through Activation of p53 Family Members

The inactivation of p53, a tumor suppressor protein, is a key event in the pathogenesis of many cancers. Numerous studies have shown that restoration of p53 function leads to the cell cycle arrest and apoptosis in cancer cells. Results of several *in vivo* and *in vitro* studies have shown that withaferin-A not only increases the expression of p53 protein but also induces the phosphorylation of p53 at serine 315 residue, thereby enhancing p53-mediated transcription of cell cycle inhibitor p21 protein in MCF-7 cells [73,74]. Human papilloma virus (HPV) oncoproteins, E6 and E7, is known to inactivate p53 protein. Withaferin-A diminished the expression of E6 and E7, thereby resulting in p53 accumulation and increased expression of p53-target genes, such as p21 and Bax in human cervical cancer (Caski) cells. The induction of Bax and concomitant suppression of Bcl-2 by withaferin-A led to the cleavage of caspase-3 and PARP, and induction of apoptosis in Caski cells [22]. Knockdown of p53 attenuated withaferin-A-induced apoptosis in MCF-7 [73] and Caski [22] cells. Moreover, withaferin-A induced p53 activation *in vivo* in DMBA-induced hamsters oral squamous cell carcinomas [31] and cervical cancer xenograft tumors [22]. Bioinformatics-based molecular docking analyses also demonstrated that withaferin-A strongly binds p53 and its target gene product p21, thereby accounting for tumor cell apoptosis [80]. However, it is yet to be investigated how withaferin-A interacts with p53 and hence stabilize the expression of this tumor suppressor protein.

#### 3.4.5. Endoplasmic (ER) Stress-Mediated Induction of Apoptosis by Withaferin-A

The induction of endoplasmic reticulum (ER) stress has been shown to be involved in withaferin-A-induced apoptosis in human renal cell carcinoma (Caki) [51] and androgen-insensitive prostate cancer (PC-3 and DU-145) cells [65]. The ER stress induction by withaferin-A in Caki cells was associated with increased phosphorylation of eukaryotic initiation factor-2α (eIF-2α), splicing of ER stress-specific XBP1, elevated expression of glucose-regulated protein-78, and the activation of CHOP [51]. The abrogation of withaferin-A-induced apoptosis by RNA silencing of CHOP or the blocking of caspase-4 activity suggests that ER stress is an alternative mechanism of apoptosis induction by this compound. Similarly, Nishikawa *et al.* demonstrated that withaferin-A induced ER stress-mediated apoptosis in PC3 and DU-145 cells as revealed by increased expression of PERK, phosphorylation of eIF-2α, activation of activated transcription factor-4 and induction of CHOP [65].

#### 3.4.6. Withaferin-A-Induced Apoptosis Is Mediated through Activation of Par-4

The activation of Par-4, a tumor suppressor protein, induces apoptosis selectively in cancer cells in a p53- or PTEN-independent manner [81,82]. Par-4 is constitutively expressed in normal rat and human cholangiocytes, but its expression declines in human cholangiocarcinoma. Whether intrahepatic or extrahepatic origin, Par-4 expression in cholangiocarcinoma was negatively correlated with cell proliferation markers but positively linked with apoptotic markers. Silencing of Par-4 by small interfering RNAs increased the proliferation of cholangiocarcinoma cells in culture. Treatment with withaferin-A induced Par-4 expression in human cholangiocarcinoma cells and inhibited cell proliferation and induced apoptosis [83]. Likewise, withaferin-A induced Par-4-dependent apoptosis in androgen-refractory, but not androgen-responsive, prostate cancer cells and the regression of PC-3 cells xenograft tumors in nude mice. However, when combined with anti-androgens, withaferin-A showed synergistic effect on Par-4-mediated apoptosis induction in androgen-responsive prostate cancer cells [66]. According to this study, withaferin-A-induced Par-4 protein caused inactivation of the NF-κB activity and the attenuation of Bcl-2 protein expression. In contrast, a recent study reported that withaferin-A treatment did not alter the mRNA and protein level of Par-4 in PC3 cells [65]. A molecular resolution of this discrepancy in Par-4 regulation by withaferin-A, and its impact on cell survival merits further investigation in different cancer histotypes.

#### 3.4.7. Other Mechanisms of Apoptosis Induction by Withaferin-A

Withaferin-A reduced the viability of soft tissue sarcoma cells, such as that of fibrosarcoma (HT1080), leiomyosarcoma (SKLMS1), STLS26T, and high grade pleomorphic sarcoma/liposarcoma through caspase-mediated degradation of vimentin, downregulation of Akt phosphorylation and the NF-κB (p65) activation [16]. Although transfection of STS cells with vimentin siRNA abrogated the inhibitory effect of withaferin-A on Akt phosphorylation and p65 activation, it remains elusive how proteasomal degradation of vimentin leads to inhibition of Akt/NF-κB signaling in these cells.

The induction of apoptosis in MCF-7 cells by withaferin-A resulted from the downregulation of estrogen receptor-α (ERα)-mediated signaling through proteasomal degradation of ERα. Withaferin-A attenuated nuclear localization and the transcriptional activity of ERα, thereby decreasing the expression of ER-α-regulated gene product pS2. This inhibitory effect of withaferin-A on ERα signaling was markedly reversed in the presence of esteradiol [73]. Withaferin-A-induced apoptosis and attenuation of ERα in these cells were associated with down-regulation of RE arranged during Transfection (RET) tyrosine kinase and heat shock factor-1 (HSF1), and up-regulation of p53 and p21 protein expression [74].

### 3.5. Autophagy Induction by Withaferin-A and Its Implication in Cancer

Autophagy, a physiological process of destroying cellular macromolecules and organelles, is basically a cell survival mechanism. However, the induction of autophagy can also lead to cell death [84,85]. Although, the precise mechanism of autophagy outcomes in terms of survival or apoptosis is still unclear, a large pool of data indicates that many anticancer agents induce autophagy as their mechanisms of action [85]. Withaferin-A has been reported to induce autophagy in human breast cancer (MDA-MB- 231 and MCF-7) as well as immortalized and non-tumorigenic MCF-10A cells [86]. Treatment with withaferin-A also induced the expression of autophagy markers in MDA-MB-231 cells xenograft tumors *in vivo*. Withaferin-A-induced apoptosis remained unaffected even after pharmacological inhibition or genetic repression of autophagy, suggesting that autophagy induction by the compound is not associated with its apoptosis-inducing ability [86]. However, Fong *et al*. [87] reported that withaferin-A treatment sensitized ovarian cancer cells to doxorubicin by inducing ROS-dependent autophagy *in vitro* and *in vivo*. Likewise, the DNA microarray of PC3 cells revealed that withaferin-A upregulated the mRNA level of Bcl-2-associated athanogene 3 (BAG3) [65], which is a member of the BAG co-chaperone family and has been implicated in autophagy [76]. Moreover, withaferin-A induced the expression of LC3, a marker of autophagy. Although withaferin-A-induced LC3 expression remained unaffected upon treatment of cells with pharmacological inhibitors of canonical autophagy, silencing of BAG3 led to suppression of withaferin-A-induced LC3 expression and cell death, suggesting that the compound induced BAG3-mediated autophagy that protects PC3 cells against withaferin-A-induced apoptosis. It would, thus, be interesting to further delineate the role of withaferin-A-induced autophagy in cancer.

### 3.6. Withaferin-A as an Inhibitor of Tumor Angiogenesis

The development of anticancer therapies by targeting the tumor angiogenesis process is a well-established drug discovery approach. The decrease in vascular endothelial growth factor (VEGF)-induced tube formation by HUVECs and attenuation of blood vessel formation in chick chorioallantoic membrane (CAM) assay by withaferin-A suggest the ability of the compound to suppress tumor angiogenesis. Treatment of Ehrlich ascites tumor-bearing mice with withaferin-A resulted in decreased peritoneal angiogenesis, microvessel density, and reduced volume of ascites tumor, partly through downregulation of VEGF production [88]. Withaferin-A inhibited the binding of Sp1 transcription factor to VEGF-gene promoter, thereby exhibiting antiangiogenic activity [88,89]. According to an *in silico* docking study, withaferin-A was found to interact with VEGF at relatively low binding energy compared to Bevacizumab [90]. Incubation of human vascular endothelial cells cultured in soft tissue sarcoma conditioned medium with withaferin-A induced apoptosis and inhibited the migration of endothelial cells. Moreover, the growth of leiomyosarcoma (SKLMS1) and fibrosarcoma (HT-1080) xenograft tumors in SCID mice was suppressed by treatment with withaferin-A, which significantly decreased tumor-associated blood vessels as revealed by the presence of small collapsed CD31-positive vessels in treated tumors [16]. A recent study also demonstrated that withaferin-A markedly reduced the expression and release of VEGF in cultured HUVECs and inhibited VEGF-induced tube formation [91], suggesting that the compound can suppress tumor angiogenesis.

### 3.7. Anti-Migratory, Anti-Invasive and Anti-Metastatic Effects of Withaferin-A

The activation of epithelial-to-mesenchymal transition (EMT) helps tumor cells in acquiring migratory and invasive properties, thereby enhancing tumor metastasis. Several studies have demonstrated the inhibitory effects of withaferin-A on the migration, invasion and metastasis of various cancers. Withaferin-A showed partial inhibition of TNFα- or transforming growth factor-β1 (TGF-β)-induced EMT and the migration of MCF-10A cells [92]. Knockdown of E-cadherin in these cells modestly abrogated the inhibitory effects of withaferin-A on TNF-α and TGF-β-induced cell migration. Whereas treatment of MCF-7 and MDA-MB-231 cells with withaferin-A elevated the level of E-cadherin protein, the expression of vimentin was decreased in the MDA-MB-231 cells xenografts as well as in MMTV-neu tumors from withaferin-A-treated mice. These findings suggest that withaferin-A inhibited experimental EMT in MCF-10A cells and mammary cancer growth inhibition partly through inhibition of vimentin protein expression *in vivo* [92]. Thaiparambil *et al*. reported that withaferin-A retained potent anti-invasive activity at sub-cytotoxic doses that induced perinuclear vimentin accumulation followed by rapid vimentin depolymerization. Moreover, a concomitant phosphorylation of vimentin at serine-56 residue and subsequent disassembly of vimentin upon treatment with withaferin-A was also noted. Withaferin-A also showed dose-dependent inhibition of metastatic lung nodules and increased vimentin phosphorylation at serine-56 in a mouse model of breast cancer *in vivo* [20].

Treatment with withaferin-A resulted in marked inhibition of the TGF‑β‑induced migration and invasion of human cervical cancer (Caski) cells by blocking the expression and the activity of matrix metalloproteinase (MMP)-9 through inhibition of Akt phosphorylation [93]. Withaferin-A alone or in combination with withanone, another withanolide present in W. somnifera, attenuated migration, invasion and *in vivo* lung metastasis of fibrosacoma (HT1080) cells by disrupting the interaction between a multifunctional RNA binding protein, heterogeneous nuclear ribonucleoprotein-K (hnRNP-K) and single stranded DNA (ssDNA). Withaferin-A directly interacted with residues Gly26, Ser27, Gly30, Lys31, Gln34, Gln83 and Ser85 of KH3 domain of hnRNP-K via hydrogen bonds and hydrophobic interactions, thereby masking the ability of hnRNP-K to bind with ssDNA. The inhibition of hnRNP-K binding with ssDNA by withaferin-A resulted in decreased expression of hnRNP-K downstream effectors, such as MMP2, pERK and VEGF, which partly accounts for the anti-migratory and anti-metastatic effect of the compound [91]. Withaferin-A strongly decreased the invasion of MDA-MB-231 cells in association with decreased expression of extracellular matrix-degrading proteases, cell adhesion molecules, pro-inflammatory mediators of the metastasis-promoting tumor microenvironment, and concomitant increased expression of breast cancer metastasis suppressor gene (BRMS1) [94]. Treatment of mice bearing orthotopic ovarian tumors generated by injecting human ovarian epithelial cancer cell line (A2780) with withaferin-A attenuated tumor growth and completely inhibited metastasis of these tumors [23].

The modulation of Notch signaling pathway is another molecular target of withaferin-A. While the compound attenuated the activation of Notch-1 in colon cancer and human breast cancer cells, it activates the cleavage of Notch-2 and Notch-4 in human breast cancer (MDA-MB-231) cells. Withaferin-A-mediated inhibition of MDA-MB-231 cell migration was significantly augmented by knockdown of Notch-2 and Notch-4 protein, suggesting that the Notch-2 and Notch-4 activation by withaferin-A impairs its inhibitory effect on breast cancer cell migration [95]. Further studies would clarify the exact role of withaferin-A in modulating various Notch isoforms and their impact on tumor progression.

### 3.8. Chemosensitizing and/or Synergistic Effect of Withaferin-A with Chemotherapeutic Agents

Cancer cells often acquire resistance to chemotherapy and radiotherapy. Many chemopreventive phytochemicals act as adjunctive therapy to alleviate chemoresistance and radioresistance, thereby suppressing tumor growth. One of the mechanisms underlying chemoresistance is the overexpression of P-glycoprotein (P-gp), a drug efflux protein that is induced via transcriptional activation of NF-κB, AP1 and Nrf-2 transcription factors, and pumps the chemotherapeutic drugs out of the cells. Besides its drug efflux property, P-gp also plays a specific role in blocking caspase-dependent apoptosis. Treatment of doxorubicin-resistant human leukemia cells (K562/Adr) with withaferin-A resulted in caspase-mediated apoptosis induction, which was associated with decreased expression of Bcl-2, Bim and p-Bad. Interestingly, withaferin-A in these cells reduced the expression of tubulin via a direct thiol oxidation mechanism [96]. Treatment of various ovarian cancer cell lines (A2780, A2780/CP70 and CaOV3) with a combination of withaferin-A and doxorubicin elicited synergistic antiproliferative and apoptosis-inducing effects, thus reducing the requirement of lower chemotherapeutic dose of doxorubicin and minimizing doxorubicin-induced adverse drug reactions [87]. This combination approach resulted in a significant increase in ROS production resulting in DNA damage, induction of autophagy as reveled by increased expression of autophagy marker LC3B, and induced cell death via caspase-3 cleavage. Moreover, withaferin-A synergistically caused a 70%–80% reduction in the growth of ovarian tumor cells xenograft in nude mice [87]. Kakar *et al.* also demonstrated that withaferin-A sensitizes ovarian cancer (A2780) cells to cisplatin-induced cytotoxicity. According to their study, a regimen comprising withaferin-A and a suboptimal dose of cisplatin induced cell death through the generation of ROS [97]. In a subsequent study, these authors further reported that withaferin-A alleviated cisplatin resistance largely by eliminating cells expressing various cancer stem cell markers [23].

### 3.9. Targeting Cancer Stem Cells with Withaferin-A

Cancer stem cells are a small population of tumor-initiating cells with self-renewal capacity. The increasing trend of chemotherapy failure and recurrence of cancer has been attributed to the existence of cancer stem cells in growing tumor. Thus, eradication of cancer stem cells has been a priority in recent endeavors of anti-cancer drug development. Su *et al.* reported that the overexpression of nestin, a stem cell marker, in pancreatic ductal adenocarcinoma cells increased cell motility and induced phenotypic changes associated with the EMT, whereas knockdown of endogenous nestin expression reduced cell migration and helped cells to retain epithelial phenotype. The inhibitory effect of withaferin-A on the anti-nestin activity in these cells suggests that the compound holds the potential to target pancreatic cancer stem cells [98]. In another study [99], withaferin-A dose-dependently inhibited *in vitro* mammosphere formation, the aldehyde dehydrogenase 1 (ALDH1) activity, and CD44^high^-CD24^low^-epithelial-specific antigen-positive (ESA+) fraction in cultures of MCF-7 and SUM159 human breast cancer cells. In addition, administration of withaferin-A to MMTV-neu mice resulted in the inhibition of mammosphere number and the ALDH1 activity *in vivo*. Withaferin-A resulted in a cell line-specific alteration in the mRNA expression of cancer stem cell markers, such as Oct4, SOX-2, and Nanog. Overexpression of urokinase plasminogen activator (uPA), a factor involved in attaining the stemness [100], increased the number of MCF-7 cells mammosphere, which was partially inhibited by treatment with withaferin-A. Moreover, withaferin-A treatment reduced the level of Bmi (B-cell-specific Moloney murine leukemia virus insertion region-1) protein, which has been implicated in the self-renewal of breast cancer stem cells [101], and reduced the ALDH1 activity in Bmi-overexpressing MCF-7 cells. This study also revealed that the inhibitory effect of withaferin-A on breast cancer stem cells proliferation was partly mediated through cleavage of Notch-4 [99]. The antitumor effects of withaferin-A alone or in combination with cisplatin in mice bearing ovarian tumors were attributed to the elimination of cells expressing cancer stem cells markers, such as CD44, CD24, CD34, CD117 and Oct4, and the downregulation of Notch-1, Hes1 and Hey1 genes [23].

### 3.10. Withaferin-A as a Cancer Immunotherapy

*In vitro* treatment with withaferin-A decreased the production of IL-10 by myeloid-derived suppressor cells (MDSC), a class of regulatory T cells that inhibit activation and tumor infiltration of cytotoxic T cells. The compound also reduced the secretion of IL-6 and TNFα, which increases MDSC accumulation and function, from tumor-associated macrophages (TAM). The T-cell suppressive activity of MDSC is due to the production of ROS, which was significantly inhibited by withaferin-A in a STAT3-dependent mechanism. Moreover, *in vivo* treatment of tumor-bearing mice with withaferin-A decreased tumor weight, reduced the number of granulocytic MDSC, and blocked MDSC-mediated suppression CD4+ and CD8+ T cells activation. These findings suggest that withaferin-A may be used as adjunctive cancer immunotherapy to facilitate the anti-tumor immunity [102]. It is interesting to note that a wide variety of cancer immunotherapies are emerging, but many of them suffer from unwanted side effects from overactivation of CD8+ T cells-mediated adaptive immune response. A recent study demonstrated that withaferin-A inhibited mitogen-induced T-cell and B-cell proliferation *in vitro* and reduced the secretion of Th1 and Th2 cytokines. This immunosuppressive effects of withaferin-A was mediated through its binding with cysteine-62 residue of p50 and subsequent inactivation of the NF-κB pathway in T lymphocytes [103]. While the MDSC suppressive effect of withaferin-A can enhance tumor specific T cell activation, this peripheral immunosuppression by the compound indicates that it may be selected as an ideal candidate for further development as a cancer immunotherapy.

## 4. Withaferin-A as a Cysteine Thiol Modifier

A great deal of research has been done to explore the molecular details of antitumor effects of withaferin-A. Several studies have shown that withaferin-A can directly bind with a number of intracellular signaling molecules and alter cell fate, such as proliferation, migration and invasion. The inappropriate activation of NF-κB drives the oncogenic signaling and contributes to attaining different hallmarks of cancer. Withaferin-A inhibited NF-κB activation by preventing TNFα-induced activation of IκB kinase-β (IKKβ) via a thioalkylation-sensitive redox mechanism, thereby blocking phosphorylation and subsequent degradation of IκB, and suppressing nuclear translocation and DNA binding of NF-κB [104]. Grover *et al*. reported that withaferin-A formed strong intermolecular interactions with the NEMO (NF-κB Essential Modulator) chains and imposed steric as well as thermodynamic hindrance to the incoming IKKβ subunits, thereby suppressing NF-κB activation [45]. Heyninck *et al*. recently demonstrated that withaferin-A inhibited catalytic activity of IKKβ, a kinase which is indispensable for the nuclear translocation of NF-κB, by directly interacting with cysteine-179 residue IKKβ. Docking of withaferin-A to a IKKβ homology structure model showed that the compound fit nicely into the groove of IKKβ where it can form hydrogen bond to stabilize its interaction with cysteine-179. Mutation of cysteine-179 abrogated the NF-κB suppressing effect of withaferin-A [46]. However, a recent study reported that withaferin-A did not disrupt RIP1 polyubiquitination or NEMO-IKKβ interaction, and was a poor direct IKKβ inhibitor in the canonical pathway induced by TNF-α. According this study, withaferin-A blocked the formation of TNF-induced NEMO foci which colocalized with TNF ligand. The compound inhibited IKK function by disrupting NEMO reorganization into ubiquitin-based signaling structures [105]. As has been mentioned earlier, withaferin-A abolished NF-κB activation in T lymphocytes by covalent modification of cysteine-62 of p50 subunit of NF-κB [103].

Alteration of microtubule and microfilament function occurs during aberrant cell proliferation and migration of cancer cells. Withaferin-A caused direct covalent binding with cysreine-302 residue of β-tubulin, thereby inducing cell cycle arrest in MCF-7 cells [64]. Since annexin-A2 is a critical regulator of migration, invasion and angiogenesis processes [106], the thiol modification of this protein appears as a novel mechanism of anticancer activity of withaferin-A. Falsey *et al.* [107] first demonstrated that withaferin-A covalently interacted with cysteine-133 residue of annexin-A2, a Ca^2+^ binding protein integrated into a network of cellular processes including actin bundling, thereby stimulating F-actin cross-linking. In contrast, withaferin-A has been shown to bind with the cysteine-9, but not the cysteine-133, residue located on the N-terminal domain of annexin-A2 [108]. This study has suggested that the increase of F-actin bundling activity and the decrease in actin polymerization are possible mechanisms by which withaferin-A binding may play roles in Anxnexin A2-mediated cellular actin dynamics [108]. Further studies are necessary to delineate the role of withaferin-A in modulating actin polymerization. Withaferin-A treatment of human fibroblasts rapidly reorganizes vimentin intermediate filaments into a perinuclear aggregate. Although the cysteine-328 of vimentin has been proposed as a putative binding site for withaferin-A, its cellular consequences are unclear [109]. Based on these reports, it can be concluded that withaferin-A is a potent thiol modifier and can interfere with diverse signaling pathways implicated in oncogenic processes.

## 5. Conclusions

A wide variety of plant secondary metabolites have gained the reputation of anticancer agents. Over the years, these phytochemicals are being used either as part of our regular diet or in the form of Ayurvedic medicine and have proven their safety. Interestingly, many of these anticancer phytochemicals are multi-targeted in modulating abnormal intracellular signaling generally driving the carcinogenic processes. Since cancer is a systemic multifactorial disease, multi-targeted agents rather than a single gene-targeting therapy would be an appropriate choice in eradicating cancer. Systematic research on Ashwagandha and its major bioactive metabolite, withaferin-A, has revealed the potential of this withanolide to elicit multi-targeting effects on various cancer hallmarks. Despite substantial progress made in understanding the mechanisms underlying the anticancer effects of withaferin-A, there is a dearth of knowledge about the effect of the compound on restructuring the tumor microenvironment to disrupt the pro-tumorigenic niche existing between stromal, immunoinflammatory and tumor cells. Moreover, detailed pharmacokinetic studies to ascertain the biologically active dose of the compound and its disposition pattern need further work. Nonetheless, evidence from current preclinical studies suggests withaferin-A to be a promising candidate for further development as an anticancer drug.

## Figures and Tables

**Table 1:** Molecular mechanisms underlying anti-proliferative and apoptosis inducing activity of withaferin-A.

Experimental Models	Molecular Targets	Ref.
**Prostate cancer cells**	Induced G2/M phase arrest. ↑ phosphorylation of Wee-1, p21, Aurora B and histone H3 ↓ expression of cyclin-A2, -B1 and -E2 ↓ phosphorylation of Cdc2, (tyrosine-15), Chk1 (serine-345) and Chk2 (threonine-68)	[58]
	↑ generation of ROS ↑ mRNA and protein expression of c-Fos ↓ expression of c-FLIP; Disruption of vimentin subcellular localization	[65]
	↑ Par-4 expression ↓ NF-κB activity ↓ Bcl-2 expression	[66]
**Osteosarcoma cells (MG63 and U20S)**	Induced G2/M phase arrest. ↑ phosphorylation of Chk1 (ser345) and Chk2 (Thr68) ↓ phosphorylation of Cdc2 (tyrosine-15) ↓ expression of cyclin-A and -B1, and Cdk2	[57]
**Ovarian cancer cells (CaOV3 and SKOV3)**	Induced G2/M phase arrest. ↓ expression of Notch-3 and Bcl-2 ↓ phosphorylation of Akt ↑ activation of caspase-3 and cleavage of PARP	[60]
**Colorectal cells (HCT116 and SW480)**	Induced G2/M phase arrest. ↑ proteasomal degradation of Mad2 and Cdc20; interference with spindle assembly and delayed mitosis	[55]
**Lung cancer cells (A549)**	Induced G0/G1 phase arrest. ↓ expression of Bcl-2 ↓ phosphorylation of Akt ↑ activation of caspase-3 and cleavage of PARP	[62]
**Head and neck carcinoma cells (MC3 and HN22)**	↑ expression of Bim, t-Bid, c-caspase-8, DR-5, c-PARP	[67]
**Human renal carcinoma cells (Caki)**	↑ ROS generation ↑ expression of CHOP and DR-5 ↓ activation of NF-κB and expression of c-FLIP	[68]
**Glioblastoma multiforme cells**	↑ expression of cyclin B1 and induction of G2/M phase arrest ↓ phosphorylation of Akt, mTOR, p70 S6K ↓ expression of c-Met, EGFR and Her2 ↑ activation of caspase-8,-9, -7 and -3 ↑expression of HSP70 and HSP32 ↓ expression of HSF-1	[56]
**Human leukemia cells (HL60)**	↑ generation of ROS ↑ activation of caspase-9 and -3 ↑ mitochondrial localization of Bax and release of cytochrome c ↑ cleavage of PARP ↑ caspase-8 cleavage ↓ expression of Bid ↓ activation of NF-κB	[69]
**Melanoma cells**	↑ generation of ROS ↑ activation of caspase-9 and -3 ↓ Bcl-2/Bax and Bcl-2/Bim ratios	[70]
**Cervical cancer cells (Caski)**	Induces G2/M phase arrest. ↓ expression of E6/E7 ↑ accumulation of p53,and expression of p21 and Bax ↓ expression of cyclin B1, Cdc2, Bcl-2 and PCNA ↓ phosphorylation of STAT3	[22]
	↓ GSH/GSSG ratio ↑ caspase-3 activation ↑ tBid and Noxa expression ↓ Bcl-2 and Mcl-1 expression	[71]
**Breast cancer cells**	↑ expression of Bim-S and Bim-EL ↑ cleavage of PARP ↓ expression of Bcl-2	[19]
	↓ expression of XIAP; cIAP2 and survivin	[72]
	↓ expression of ERα and pS2 ↑ expression of p53 and p21 ↑ phosphorylation of serine-315 of p53	[73,74]
	Binding to cysteine-303 of β-tubulin and ↓ Hsp90 activity	[64]
	↑ generation of ROS ↑ activation of Bax and Bak	[75]
	↑ autophagy ↑ expression of LC3 and BAG3	[76]
**Pancreatic cancer cells**	↓ Hsp90 activity ↓ expression of Akt, Cdk4 and glucocorticoid receptor	[32]
